# NAD^+^ oscillation and hypothalamic neuronal functions

**DOI:** 10.12703/r/10-42

**Published:** 2021-04-27

**Authors:** Kyohei Tokizane, Shin-ichiro Imai

**Affiliations:** 1 Department of Developmental Biology, Washington University School of Medicine, Campus Box 8103, 660 South Euclid Avenue, St. Louis, MO 63110, USA; 2 Department of Medicine, Washington University School of Medicine, Campus Box 8066, 660 South Euclid Avenue, St. Louis, MO 63110, USA; 3 Department of Gerontology, Laboratory of Molecular Life Science, Institute of Biomedical Research and Innovation, Kobe, Japan

**Keywords:** Aging, NAD^+^ oscillation, Hypothalamus, Sirtuins, NAMPT

## Abstract

A substantial body of evidence shows the importance of nicotinamide adenine dinucleotide (NAD^+^) biosynthesis and its regulation in a wide range of cellular metabolism. The expression of nicotinamide phosphoribosyltransferase (NAMPT) is regulated in a circadian manner by the core clock mechanism and NAD^+^-dependent sirtuins, producing the circadian oscillation of NAD^+^. The hypothalamus is a critical center for the homeostatic regulation of metabolism, circadian rhythm, and age-associated physiology. The dysfunction of systemic NAD^+^ biosynthesis over age affects the functions of hypothalamic neurons, causing age-associated metabolic pathophysiologies, including obesity and age-associated diseases. These recent studies suggest that NAD^+^ oscillation contributes to the hypothalamic function, and its disruption produces circadian and aging-related metabolic disorders. Furthermore, new studies have demonstrated a novel intertissue NAD^+^-dependent communication as a potential target for preventing and treating such disorders and for extending the health span of humans.

## Introduction

Over the past decade, interest in the biology of nicotinamide adenine dinucleotide (NAD^+^) has significantly increased in many different fields of biomedical research. Importantly, it has become a consensus that systemic decline in NAD^+^ levels drives the pathophysiologies of aging and determines health span and potentially life span as well. Previous studies have also demonstrated that neurons in the hypothalamus, a small region of the brain which controls essential neuroendocrine and autonomic functions throughout the body, play a critical role in the regulation of aging and longevity in mammals. It has been shown that the regulation of NAD^+^ biosynthesis at a systemic level has a significant impact on the functions of those hypothalamic neurons. Additionally, NAD^+^ levels oscillate in a circadian fashion in cells and tissues, which is indispensable for the maintenance of the circadian clock. This short review highlights recent progress in NAD^+^ oscillation and discusses a potential connection between NAD^+^ biosynthesis and neuronal functions. In particular, we focus on the role of NAD^+^ in the hypothalamus that regulates physiological homeostasis in a circadian manner and its contribution to the development and progression of age-associated disorders. Finally, we discuss the mechanisms of intertissue NAD^+^ biosynthesis as a potential therapeutic target for metabolic disorders and age-associated functional decline.

## NAD^+^ biosynthesis and oscillation 

NAD^+^ is an essential compound to maintain a variety of metabolic functions^1–3^. NAD^+^ is used in many cellular redox reactions, being reduced to NADH, and mediates many essential metabolic pathways, such as fatty acid beta oxidation, the tricarboxylic acid cycle, and glycolysis^4,5^. NAD^+^ also functions as a substrate of NAD^+^-consuming enzymes for several fundamental biochemical reactions, such as protein deacetylation/diacylation by sirtuins^6^, adenosine diphosphate (ADP)-ribosylation by poly-ADP-ribose polymerases (PARPs)^7^, and intracellular Ca^2+^ regulation by CD38/157 ectoenzymes^8^. Sterile alpha and Toll/interleukin-1 receptor motif-containing 1 (SARM1) has also been discovered as a novel NAD^+^ hydrolase^9^, depleting intracellular NAD^+^ and leading to axonal degeneration. Thus, levels of NAD^+^ need to be tightly controlled through several independent pathways.

**Figure 1.**
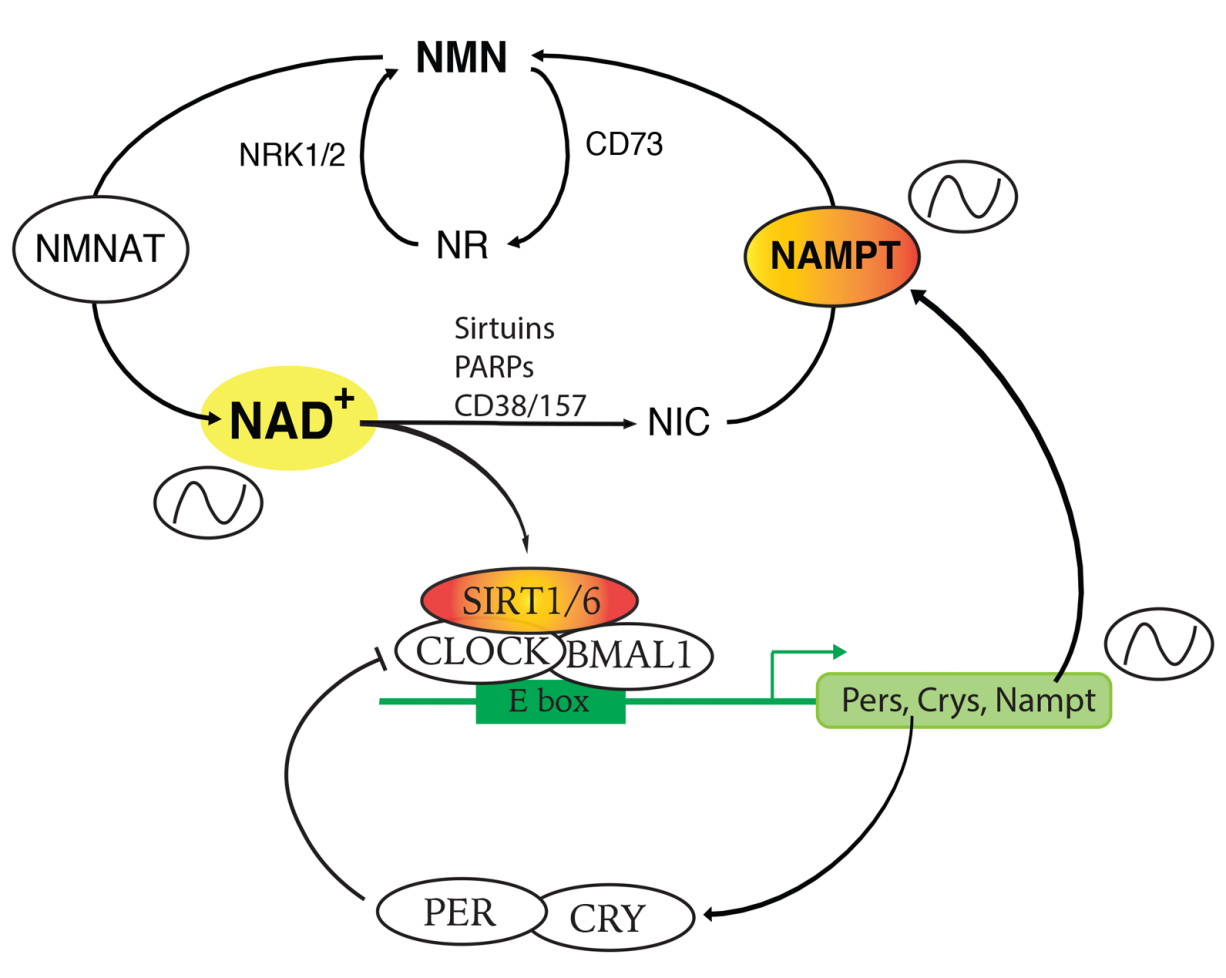
The nicotinamide adenine dinucleotide (NAD^+^) biosynthetic pathway mediated by nicotinamide phosphoribosyltransferase (NAMPT) and circadian NAD^+^ oscillation. This pathway produces a key NAD^+^ intermediate, nicotinamide mononucleotide (NMN), from a precursor nicotinamide (NIC). NMN is immediately converted to NAD^+^ by NMN adenylyltransferases (NMNATs). NIC, which is produced by NAD^+^-consuming enzymes, such as sirtuins, poly-ADP-ribose polymerases (PARPs), and CD38/157 ectoenzymes, can be salvaged into this biosynthetic pathway. NMRK1 and NMRK2 (also known as NRK1 and NRK2), as well as CD73, mediate the conversion between NMN and nicotinamide riboside (NR). Sirtuins, SIRT1 and SIRT6 in particular, modulate BMAL1/CLOCK, heterodimeric clock activators. CLOCK/BMAL1 activate various clock genes, including *period1/2*, *cry1/2*, and *Nampt*, by binding to E-boxes on their promoters. Nampt transcription shows circadian oscillation, producing circadian NAD^+^ oscillation as a metabolic oscillator. PER/CRY repress the CLOCK/BMAL1 function through protein–protein interaction, whereas SIRT1 suppresses their transcriptional activity through protein modification (deacetylation).

Five major precursors and intermediates synthesize NAD^+^: nicotinamide, nicotinamide mononucleotide (NMN), nicotinamide riboside (NR), nicotinic acid, and tryptophan. In mammals, the salvage pathway initiated from nicotinamide is dominant for intracellular NAD^+^ biosynthesis in most cells and tissues (Figure 1). Nicotinamide and 5′-phophoribose-pyrophosphate (5′-PRPP) are converted to NMN by nicotinamide phosphoribosyltransferase (NAMPT), the rate-limiting enzyme in this pathway^10^. NAMPT is an essential enzyme to regulate cellular NAD^+^ levels and is involved in a number of fundamental biological processes and disease conditions. Nicotinic acid is also used for NAD^+^ biosynthesis by nicotinic acid phosphoribosyltransferase (NAPT) in the Preiss–Handler pathway^11^, resulting in the formation of nicotinic acid mononucleotide (NaMN), which can also be derived from *de novo* NAD^+^ biosynthesis. In the *de novo* pathway, NAD^+^ is synthesized by the conversion of tryptophan to NaMN through multiple enzymatic steps^10^. NR needs to be converted to NMN by nicotinamide ribose kinases, NMRK1 and NMRK2 (also known as NRK1 and NRK2)^12^. NMN is also directly transported into cells and tissues through a newly identified transporter, solute carrier family 12 member 8 (Slc12a8), promoting NAD^+^ biosynthesis^13^.

On the other hand, NAD^+^-consuming enzymes use NAD^+^ to mediate a variety of biological processes, producing nicotinamide as a common reaction product. Nicotinamide then is recycled to produce NAD^+^ by NAMPT^14^. Thus, changes in NAMPT levels directly impact the levels of intracellular NAD^+^. Intriguingly, *Nampt* gene expression is regulated by the core circadian clock mechanism through sirtuins (Figure 1). The involvement of SIRT1, an NAD^+^-dependent sirtuin family deacetylase, in circadian regulation has demonstrated a direct link between cyclic biological rhythms and energy metabolism in the cell. SIRT1 associates with and modulates the transcriptional activity of BMAL1/CLOCK, heterodimeric clock activators^15,16^, suggesting the importance of an enzymatic feedback loop, in which SIRT1/BMAL1/CLOCK control their own activity by directing the NAD^+^ oscillation. In this regard, SIRT1 is located at a critical point where the transcriptional regulatory loop of the *Nampt* gene is connected to many biological processes through NAD^+^ as a metabolic oscillator^17^. Indeed, FK866, a highly potent NAMPT inhibitor, abolishes circadian NAD^+^ oscillation and thereby SIRT1 cyclic activity^15^. These findings have established the intimate connection between NAMPT-mediated NAD^+^ biosynthesis and SIRT1 activity in the regulation of circadian rhythm. Furthermore, NAD^+^ levels in the liver of circadian clock–deficient (*Bmal1^−/−^* or *Clock^Δ19/Δ19^*) mice were significantly reduced^15,16^, indicating that the circadian clock also controls basal NAD^+^ levels. NAD^+^ oscillation also mediates the activities of mitochondrial SIRT3 and nuclear SIRT6, which regulate mitochondrial oxidative metabolism and hepatic lipid/carbohydrate metabolism in a circadian manner, respectively^18,19^. Moreover, NAD^+^ regulates PER2 acetylation and phosphorylation to control nuclear localization of a clock repressor complex and counteracts age-associated decline in circadian functions, including dampened BMAL1 chromatin binding, transcriptional oscillations, mitochondrial respiration rhythms, and late evening activity^20^. Therefore, the circadian regulation of NAD^+^ has opened a new arena to study the molecular mechanism of physiological metabolic oscillations, linking the transcriptional feedback loop of the circadian clock to a number of NAD^+^-dependent enzymatic pathways and thereby to lifelong biological processes, such as aging.

## Role of NAD^+^ in hypothalamic neuronal functions 

The hypothalamus is a region of the brain which plays a critical role in the neuroendocrine regulation of metabolic processes and the homeostatic control of the autonomic nervous system. The hypothalamus contains several small subregions called nuclei, and each nucleus has specific functions, including the regulation of body temperature, energy intake, body fluid homeostasis, sleep, reproduction, and circadian rhythms^21–23^. For these hypothalamic functions, sirtuins, an evolutionarily conserved family of NAD^+^-dependent protein deacetylases/deacylases, have been demonstrated to be an important regulator and also a critical link to NAD^+^ metabolism^24^. For example, SIRT1 mediates central circadian control in the suprachiasmatic nucleus (SCN)^25^. Through the entrainment of cellular clocks in target tissues, the SCN coordinates the circadian control of autonomic nervous systems, neuroendocrine, and behavior^26^. In aged wild-type mice, SIRT1 levels and BMAL1 deacetylation in the SCN are decreased, causing a disrupted activity pattern, a longer intrinsic period, and an inability to adapt to the light entrainment schedule. Brain-specific SIRT1 deletion in young mice phenocopies these age-dependent circadian dysfunctions, whereas overexpressing SIRT1 in the brain counteracts the effects of aging^25^.

Mice lacking SIRT1 in proopiomelanocortin (POMC) neurons on a high-fat diet (HFD) are vulnerable to diet-induced obesity because of reduced energy expenditure even when regular chow diet (RCD)-fed conditions do not alter body weight or adiposity in these mice. The brown adipose tissue–like remodeling of the white adipose tissue through sympathetic activation requires SIRT1 in POMC neurons^27^. Likewise, deletion of SIRT1 in the steroidogenic factor 1 (SF1) neurons in the ventromedial hypothalamus (VMH) shows higher susceptibility of HFD-induced obesity whereas the mutant mice on an RCD display a normal body weight^28^. Although SIRT1 in POMC and SF1 neurons acts to resist weight gain, SIRT1 in neuropeptide Y (NPY) and agouti-related protein (AGRP) neurons is required for fasting- and ghrelin-induced hyperphagia^29^. A recent study demonstrated that central and peripheral NAD^+^ administration suppresses fasting-induced hyperphagia and weight gain in mice when administered in overnight-fasted mice^30^. Another report showed that four weeks of intraperitoneal NAD^+^ supplementation significantly attenuated weight gain in HFD-fed mice through the hypothalamus without any detectable side effects^31^. *Nampt* expression in the hypothalamus is also affected by HFD feeding or the administration of leptin and ghrelin. Moreover, inhibition of NAMPT by intracerebroventricular FK866 administration diminishes food intake during the dark period and ghrelin-induced hyperphagia^32^. Although these findings are somewhat contradictory, they suggest that NAMPT-mediated NAD^+^ biosynthesis in the hypothalamus drives metabolism and feeding behavior. Given the importance of NAD^+^ biology in the hypothalamus, further studies will be required in each individual hypothalamic nucleus or neuronal subpopulation to understand the NAD^+^-dependent regulation of central metabolic pathways.

Remarkably, brain-specific SIRT1-overexpressing (BRASTO) transgenic mice have extended median and maximal life span in both males and females^33^. Associated with this life-span extension, BRASTO transgenic mice have maintained higher body temperature, oxygen consumption, and physical activity and better sleep quality, similar to young mice, during aging. In particular, their skeletal muscle shows a youthful morphology of mitochondria and improved mitochondrial gene expression profiles during aging. These effects are most likely mediated by the activation of the sympathetic nervous system during the dark period, which may also drive other systemic events contributing to the delay in aging and life-span extension. In the hypothalamus, SIRT1 mediates neuronal activation in the dorsomedial hypothalamus (DMH) and the lateral hypothalamus (LH) through the interaction with Nk2 homeobox 1 (NKX2-1), an Nk2 family homeodomain transcription factor, and subsequent upregulation of orexin type 2 receptor (*Ox2r*) expression. Indeed, DMH/LH-specific *Sirt1* knockdown and DMH-specific SIRT1 overexpression have demonstrated the importance of the SIRT1/NKX2-1/OX2R-mediated signaling pathway in counteracting age-associated physiological decline in physical activity, body temperature, and quality of sleep^33^. Therefore, the DMH and possibly the LH are likely to be key regions of the hypothalamus which control aging and longevity in mammals.

Sirtuins are also critical for hypothalamic inflammation, which now is regarded as one of the key mechanisms underlying hypothalamic neuronal dysfunction in obesity and aging. Obesity and aging are associated with the activation of microglia and inflammatory signaling pathways in the hypothalamus^34^. Microglial nuclear factor kappa B (NF-κB) activation induces tumor necrosis factor alpha (TNF-α) production, which further impairs functions of hypothalamic neurons by activating their NF-κB in the mediobasal hypothalamus and leads to the decline in neurogenesis, bone mass, muscle strength and size, skin thickness, and life span^35^. Because NAMPT and NAD^+^ levels are reduced by TNF-α^36^ and also because SIRT1/6 and SIRT2 suppress inflammation and neurotoxicity through NF-κB deacetylation and inhibition in neurons and microglia^37–39^, microglia-mediated inflammation could suppress NAD^+^ biosynthesis in the hypothalamus and cause age-associated functional decline.

Although the physiological significance of NAD^+^ oscillation in the hypothalamus has not yet been directly tested, it is conceivable that NAD^+^ oscillation has an important role in the regulation of hypothalamic functions and also in age-related pathophysiological disorders. Indeed, in the SCN, *Nampt*, *Pgc-1α*, and *Sirt1* mRNA levels show diurnal oscillation^25^. Because SIRT1 and PGC1-α cooperatively bind to the *Bmal1* promoter and contribute to its oscillatory expression pattern^25^, NAD^+^ oscillation, which is likely produced by the oscillation of *Nampt* expression, probably contributes to the generation of SIRT1 activity oscillation in the SCN. Additionally, because tissue NAD^+^ levels significantly decrease during aging^40^, the amplitude of NAD^+^ oscillation is reduced so that the oscillation of clock genes gets attenuated even in the hypothalamus during aging, causing the derailment of circadian functions mediated by the hypothalamus. Thus, treatments that could maintain hypothalamic NAD^+^ oscillation could improve patterns of clock gene expression and ameliorate hypothalamic dysfunctions as a potential preventive/therapeutic intervention strategy for aging-related metabolic disorders.

## Mechanisms of intertissue NAD^+^ biosynthesis as a novel concept for understanding energy metabolism, circadian rhythm, and aging/longevity 

**Figure 2.**
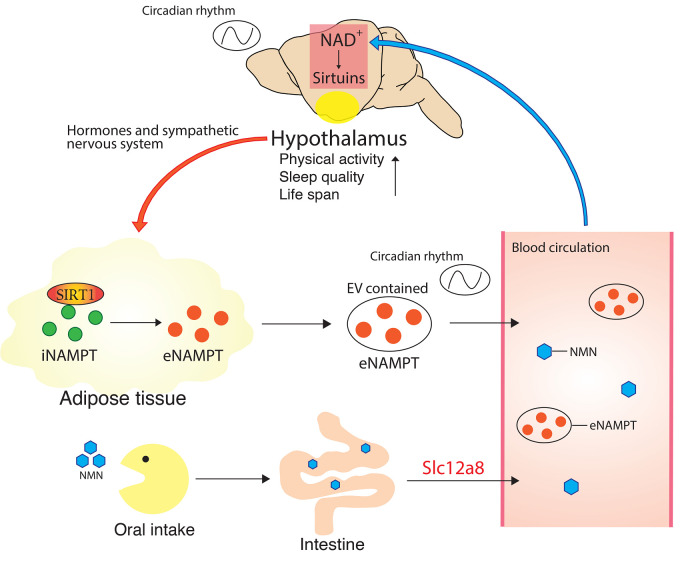
Intertissue nicotinamide adenine dinucleotide (NAD^+^)-dependent communications between the brain and peripheral tissues. The brain, especially the hypothalamus, regulates peripheral tissues, such as adipose tissue, through hormones and the sympathetic nervous system. Nicotinamide phosphoribosyltransferase (NAMPT) is secreted from adipose tissue as the extracellular vesicle (EV)-contained extracellular form (eNAMPT) into the blood circulation. EV-contained eNAMPT is internalized into neurons in the brain, which produces nicotinamide mononucleotide (NMN) intracellularly and eventually enhances NAD^+^ biosynthesis. The oscillation of circulating eNAMPT could remotely enhance NAD^+^ oscillation and thereby neuronal functions in the hypothalamus, regulating neuroendocrine function, sleep, circadian rhythm, and aging/longevity. NMN is transported into the blood circulation via Slc12a8, a newly identified NMN transporter, in the small intestine, and NMN could modulate brain functions through the generation of NAD^+^ and the activation of sirtuins. Another NAD^+^ precursor, nicotinamide riboside, may also modulate brain functions pharmacologically. iNAMPT, intracellular nicotinamide phosphoribosyltransferase.

According to recent studies on the systemic regulation of NAD^+^ biosynthesis, hypothalamic NAD^+^ biosynthesis appears to be modulated by circulating NAD^+^ intermediates and/or extracellular NAMPT (eNAMPT) secreted from peripheral tissues (Figure 2). Our own studies have shown that NMN circulates in blood circulation in mice^41^ and that plasma NMN levels decrease during aging^13,42^. When given through gavage or intraperitoneal injection, NMN can enter the blood circulation very quickly (within several minutes) and start increasing tissue NAD^+^ levels within 15 minutes. For example, NMN can efficiently increase hippocampal and hypothalamic NAD^+^ levels^43,44^. The mechanism that facilitates such a quick transport of NMN remained unknown until recently. In 2019, our group discovered that the *Slc12a8* gene encodes a novel NMN transporter and is highly expressed in the mouse small intestine^13^. The *K_m_* of Slc12a8 for NMN is 34 μM, which is consistent with measured NMN concentrations in mouse plasma. As mentioned above, NAD^+^ levels decrease in multiple tissues during aging, and the small intestine is not an exception. In particular, the aged ileum shows decreased NAD^+^ levels, and *Slc12a8* expression is upregulated as a compensatory response. When this upregulation was suppressed, the aged ileum failed to maintain its NAD^+^ levels, suggesting that the NMN-transporting function of Slc12a8 is critical to counteract age-associated NAD^+^ decline in the small intestine. Because Slc12a8 is also expressed in the hypothalamus and other brain regions (our unpublished finding), it is conceivable that NMN uptake through Slc12a8 plays an important role in regulating NAD^+^ levels in the hypothalamus. Interestingly, NMN is contained in certain vegetables and fruits, such as edamame, broccoli, cucumber, avocado, and tomato^45^. NR is also contained in human^46^ and cow^47^ milk. Furthermore, although it has been reported that NR completely degrades to nicotinamide, which then is converted to nicotinic acid in the intestine^48^, it has also been reported that orally administrated NR increases NAD^+^ in the hypothalamus without changing nicotinamide in mice^20^. These recent findings suggest that orally administered NAD^+^ intermediates, even from natural food, potentially boost hypothalamic NAD^+^ biosynthesis.

eNAMPT is secreted mainly from adipose tissue through SIRT1-mediated deacetylation of intracellular NAMPT (iNAMPT)^43^. Intriguingly, adipose tissue–specific *Nampt* knockout (ANKO) mice exhibit reduced eNAMPT levels in blood circulation, causing decreases in hypothalamic NAD^+^ and SIRT1 activity and their physical activity^43^. Furthermore, aged adipose tissue–specific *Nampt* knockin (ANKI) mice exhibit youthful levels of eNAMPT in their plasma and higher levels of NAD^+^ in multiple tissues, including the hypothalamus, and maintain improved levels of physical activity, glucose metabolism, cognitive function, and sleep quality^49^. The circulating levels of eNAMPT significantly decline with age in mice and humans. Remarkably, in both humans and mice, eNAMPT is contained exclusively in extracellular vesicles (EVs) in the blood circulation. Primary hypothalamic neurons can uptake EV-contained eNAMPT, but not eNAMPT alone, and internalized eNAMPT can promote NAD^+^ biosynthesis. Consistent with this finding, the administration of eNAMPT-containing EVs purified from young mice or cultured adipocytes promotes wheel-running activity and extends the life span in aged mice. Because EVs purified from *Nampt*-knockdown adipocytes fail to enhance wheel-running activity, eNAMPT is responsible for this aging-counteracting effect^49^. Thus, this EV-mediated delivery of eNAMPT comprises a new intertissue communication between the hypothalamus and adipose tissue, which is critical for maintaining systemic NAD^+^ biosynthesis and counteracting age-associated physiological decline.

Many studies have reported that circulating eNAMPT levels increase in obesity and type 2 diabetes^3^. Interestingly, it has been reported that a monomeric form of eNAMPT increases in HFD-induced type 2 diabetes model mice and also that the administration of monomeric eNAMPT induces diabetic phenotypes in mice^50^. A follow-up study from the same group demonstrated that under normal physiological conditions, eNAMPT maintains a dimeric form and enhances pancreatic beta-cell function through an NAD^+^-dependent mechanism whereas eNAMPT at much higher concentrations tends to be converted to a monomeric form, inducing beta-cell dysfunction through an NAD^+^-independent pro-inflammatory mechanism^51^. Given that eNAMPT is encapsulated exclusively in EVs and does not cause any metabolic dysfunction under physiological conditions^49^, it is of great interest to examine what conditions promote the conversion of eNAMPT from dimeric to monomeric forms and where monomeric eNAMPT comes from. An alternative possibility would be that the elevation of eNAMPT in obesity and type 2 diabetes is a protective mechanism that counteracts insulin resistance and stimulates beta-cell function. Thus, it is also important to further investigate the pathophysiological significance of eNAMPT in age-associated metabolic dysfunctions.

It is very likely that the maintenance of sufficient NMN/NAD^+^ levels by circulating eNAMPT is critical for tissue functions, particularly the brain and pancreatic functions, because, compared with other tissues, these tissues have very low levels of iNAMPT^41^. These findings implicate a potential fragility of pancreatic islets and central neurons in the regulation of systemic NAD^+^ biosynthesis. Interestingly, NAMPT concentrations in human serum follow a diurnal rhythm, peaking in the afternoon^52^. In mice, circulating eNAMPT levels also show a trend of diurnal rhythm, peaking during the dark period^49^. Additionally, administration of an NAMPT- neutralizing antibody suppresses NAD^+^ biosynthesis in the hypothalamus *in vivo* whereas eNAMPT enhances hypothalamic NAD^+^ biosynthesis, SIRT1 activity, and neural activity *ex vivo*^43^. These findings suggest that peripheral eNAMPT oscillation remotely modulates/enhances NAD^+^ oscillation and neuronal functions in the hypothalamus (Figure 2). Although delivery mechanisms of EV-contained eNAMPT remain unclear, eNAMPT supplementation is potentially an effective intervention for disorders related to disrupted hypothalamic NAD^+^ biology and age-associated functional decline.

## Concluding remarks

The accumulating body of evidence reveals the essential interaction between systemic NAD^+^ biosynthesis and hypothalamic neuronal functions regulating energy metabolism, circadian rhythm, and aging/longevity. NAD^+^ biosynthesis is mediated in a circadian manner, particularly by the cooperation between NAMPT and NAD^+^-consuming sirtuins. Further investigation is necessary to dissect precise details of the link between NAD^+^ oscillation and hypothalamic neuronal functions. Several key questions remain: (1) Are NAD^+^ oscillation patterns and amplitudes different in each hypothalamic nucleus or neuronal subpopulation? (2) How could the NAD^+^ oscillation in each hypothalamic nucleus potentially contribute to age-associated pathophysiologies of hypothalamic functions? (3) Do circulating eNAMPT and NAD^+^ precursors actually mediate the NAD^+^ oscillation in the hypothalamus? If so, how? As we address these questions, investigating intertissue NAD^+^-dependent communications will provide new insights into how we can better understand and manipulate the functions of the hypothalamus to ameliorate metabolic disorders and age-associated functional decline in mammals. Lastly, by elucidating what physiological conditions enhance NAD^+^ oscillation in the hypothalamus, we will be able to establish a concrete scientific foundation to develop an effective approach to prevent and treat age-associated pathophysiologies, including circadian/metabolic dysfunctions, and thereby extend the health span of humans.
